# Chemical Composition of Sour Beer Resulting from Supplementation the Fermentation Medium with Magnesium and Zinc Ions

**DOI:** 10.3390/biom10121599

**Published:** 2020-11-25

**Authors:** Aneta Ciosek, Katarzyna Fulara, Olga Hrabia, Paweł Satora, Aleksander Poreda

**Affiliations:** Department of Fermentation Technology and Microbiology, University of Agriculture in Krakow, 30-149 Krakow, Poland; k.fulara@urk.edu.pl (K.F.); o.hrabia@urk.edu.pl (O.H.); p.satora@urk.edu.pl (P.S.); a.poreda@urk.edu.pl (A.P.)

**Keywords:** sour brewing, *Lactobacillusbrevis*, aroma compounds, carbohydrate use, magnesium, zinc, wort enrichment

## Abstract

The bioavailability of minerals, such as zinc and magnesium, has a significant impact on the fermentation process. These metal ions are known to influence the growth and metabolic activity of yeast, but there are few reports on their effects on lactic acid bacteria (LAB) metabolism during sour brewing. This study aimed to evaluate the influence of magnesium and zinc ions on the metabolism of *Lactobacillus brevis WLP672* during the fermentation of brewers’ wort. We carried out lactic acid fermentations using wort with different mineral compositions: without supplementation; supplemented with magnesium at 60 mg/L and 120 mg/L; and supplemented with zinc at 0.4 mg/L and 2 mg/L. The concentration of organic acids, pH of the wort and carbohydrate use was determined during fermentation, while aroma compounds, real extract and ethanol were measured after the mixed fermentation. The addition of magnesium ions resulted in the pH of the fermenting wort decreasing more quickly, an increase in the level of L-lactic acid (after 48 h of fermentation) and increased concentrations of some volatile compounds. While zinc supplementation had a negative impact on the *L. brevis* strain, resulting in a decrease in the L-lactic acid content and a higher pH in the beer. We conclude that zinc supplementation is not recommended in sour beer production using *L. brevis WLP672*.

## 1. Introduction

Sour beer is characterised by a low ethanol content and a refreshing taste and is becoming more popular worldwide. In the production of sour beer, the main metabolite released by lactic acid bacteria (LAB) is lactic acid (LA), which creates the specific, crispy bouquet of the beer. The LA decreases the pH of the beer, which is the main factor inhibiting the growth of LAB cultures and LA production [1]. Peyer et al. [2] revealed that LAB metabolism was reduced below pH 4.9 and their growth was completely inhibited at pH below 3.4. Therefore, delaying or reducing the rate of pH drop is beneficial for the growth of LAB. This can be done by adding alkalising agents (e.g., calcium carbonate or potassium hydroxide) that react with LA to form lactate salts. This method can increase the production of LA up to eight-fold, but the produced LA must be recovered through separation [3]. Alternatively, the buffering capacity of the wort can be increased by, for example, changing the mash profile, prolonging the protein digestion or adding a citrate-based buffer [2]. In this way, it is possible to increase the production of LA by 30–40% compared to a standard wort while maintaining a relatively high pH (4.3) in the fermentation medium.

The nutritional requirements of LAB are complex and varied, even between strains within the same genera. LAB require sufficient carbohydrates, proteins, amino acids, fatty acids, vitamins and minerals for proper growth [4].

**Scheme 1:**
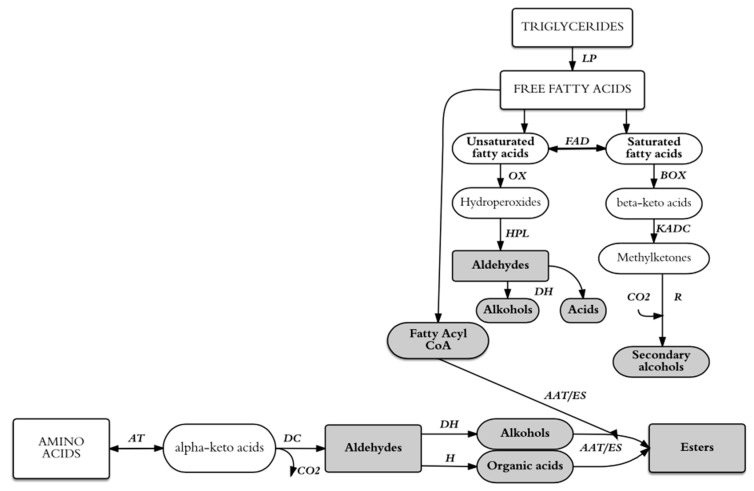
Schematic representation of main aroma compounds and organic acids that can be generated lactic acid fermentation of malt wort from existing precursors. AAT: alcohol acyltransferase; AT: aminotransferases; BOX: ß-oxidation; DC: decarboxylase; DH: dehydrogenase; ES: esterase; FAD: fatty acid desaturase; H: hydrogenase; HPL: hydroperoxide lyase; KADC: ß-ketoacyl decarboxylase; LP: lipase; OX: oxidation; R: reductase. Based on Nsogning Dongmo et al. [9].

The metabolic activity and fermentation efficiency of LAB depend on the availability of fructose [5], some amino acids (e.g., lysine, arginine, and glutamic acid) [5,6] and microelements (e.g., manganese, magnesium, potassium) [7,8]. Scheme 1 shows the potential metabolic pathways for the generation of aroma compounds from wort precursors during lactic acid fermentation.

Metal ions play important roles in many biological processes in living organisms. They are crucial for yeasts to grow and perform alcoholic fermentation. For most strains of LAB, the most important ions are manganese, magnesium, potassium and sodium [8,10]. Manganese and magnesium are crucial for the growth and metabolism of LAB; the former is important for the functioning of numerous enzymes, such as RNA polymerase, glutamine synthetase and lactate dehydrogenase [7,8], while the latter catalyses reactions of cell division, stabilises nucleic acids and is involved in protein hydrolysis [11]. Recently, potassium ions have been linked with long-range electrical signalling within bacterial biofilm communities, with potassium ions triggering depolarisation across the bacterial ecosystem [12]. LAB show no specific iron requirements and the supplementation of growth media with iron does not stimulate lactobacilli growth [11].

Malt wort has been shown to be a very good source of nutrients for yeast [1]. The microelements content is usually sufficient for microorganisms, but some of them (mainly zinc) are often replenished. Some studies have shown the role of magnesium and zinc ions in the metabolism of yeast cells [13,14] and bacteria [8,15]. In yeast, Mg^2+^ affects the permeability of cytoplasmic membranes, participates in the construction of the most important cellular structures (i.e., ribosomes, DNA and RNA) [8,15] and activates over 300 enzymes, including synthetase, phosphofructokinase, keratin kinases and membrane ATPase [16]. While zinc stimulates the protein synthesis during fermentation and is one of the key glycolytic enzymes (e.g., ethanol dehydrogenase and aldolase). So that Zn^2+^ are crucial to perform a successful fermentation. Their deficiency may result in a stuck fermentation [17]. In the past, research has revealed the requirements for metal ions by brewers’ yeast, showing the change in the uptake and release of metal ions during the fermentation process, and also how it changes with successive reuse of yeast biomass in the fermentation process [18]. It has also been revealed that ionic composition of wort has significant influence on the resistance of yeast in the stressful environment (high osmotic pressure, ethanol concentration, etc.) [17]. Previous studies on the metal ion requirements of LAB were mostly performed using milk or MRS (De Man, Rogosa and Sharpe) medium (the model growth medium) and focused on the growth of the microorganisms. In the case of LAB, the addition of magnesium ions to the culture medium was shown to reduce the growth of the bacteria [13]. However, it has been proved that the growth conditions during the propagation of *Lactobacillus* in malt wort are significantly different to those in MRS [19]. Therefore, the novelty of the work is based on the approach of evaluating the influence of metal ions on the LAB in the brewers’ wort as the culture medium.

Therefore, this study aims to evaluate the influence of the addition of magnesium and zinc ions on the metabolism of *Lactobacillus brevis WLP672* during fermentation of brewers’ wort. The rate of fermentation, pH decrease, metal ion content and the concentration of organic acids. Key volatile compounds resulting from the mixed fermentation of lactic acid bacteria and brewers’ yeast were also evaluated in the sour beer.

## 2. Materials and Methods

### 2.1. Wort

Wort (12% *w*/*w* extract content) was prepared using pale (EBC < 9), un-hopped powdered malt extract (SLADOVNA, SPOL. S ro., Bruntal, Czech Republic) and divided into five lots: two were supplemented with magnesium (Mg.1 and Mg.2) and two with zinc (Zn.1 and Zn.2), while the remaining one was used as the reference sample (REF). Magnesium sulphate (MgSO_4_·8H_2_O) was added at two different concentrations (60 mg/L and 120 mg/L), as was zinc chloride (ZnCl_2_·4H_2_O; 0.4 mg/L and 2 mg/L). All samples were sterilised in an autoclave (121 °C for 30 min) and then cooled in cold water to room temperature. Atomic absorption spectrometry was applied to check the initial concentrations of zinc and magnesium after the sterilisation (and potential precipitation). The initial magnesium and zinc ion contents of the different wort samples are shown in Table 1. The wort (50 mL) was poured into 100 mL sterile bottles and inoculated with microorganisms to perform the fermentation. Each treatment had three repeats.

### 2.2. Microorganisms and Fermentation

A culture of *Lactobacillus brevis WLP672* (White Labs Inc, Old Sarum, United Kingdom) was propagated according to a procedure described in previous research [19] (12% wort extract, temp. 25 °C, 72 h). The cultured bacteria (0.1 mL contained 6.5 × 10^7^ cells) was then added to the wort to start LA fermentation. Fermentations continued for 3 days at 25 °C under anaerobic conditions. After 72 h, the alcoholic fermentation was initiated by the addition of dry yeast (Fermentis Safale US-05, Lesaffre, France) rehydrated in water. An aliquot of rehydrated yeast slurry (0.17 mL; 5.8 × 10^8^ cells/mL) was added to each bottle to obtain an initial yeast content of 1.2 × 10^7^ cells/mL (automatic yeast counter Oculyze BB 2.0, BIOTECON Diagnostics, Potsdam, Germany). The samples were closed with a fermentation tube and then transferred into a thermostatic cabinet at 20 °C for alcoholic fermentation. At the end of fermentation, the produced sour beer samples were subjected to physicochemical analysis (ethanol content, real extract, pH, metal ions, volatile compounds, organic acids, and carbohydrates profiles).

### 2.3. Analytical Methods

#### 2.3.1. Physiochemical Analysis

A DMA 4500 M (Anton Paar, Warsaw, Poland) beer analyser was used to determine the ethanol content and real extract. Before measurement, the samples were degassed and filtered with diatomaceous earth to clarify the beer.

The pH of the beer was measured daily using a CP-411 pH meter (Elmetron, Zabrze, Poland), which was calibrated using standard buffers before each measurement was taken.

#### 2.3.2. Analysis of Metal Ions

Samples of wort and beer (3 mL) were placed in sealed pressure vessels, with the addition of nitric acid (5 mL, 68%), and subjected to wet mineralisation in a Mars Xpress microwave oven (1200 W, 170 °C, 15 min) (CEM Corp., Matthews, NC, USA). The samples were then diluted with deionised water and their absorbance at 202.6 nm for Mg^2+^ and 213.9 nm for Zn^2+^ was determined by atomic absorption spectrometry with a flame atomization technique (Varian AA240FS), using an automatic dispensing sample system (SIPS-20, Agilent, Santa Clara, CA, USA). Gas flow was 3.5 dm^3^/min (acetylene) and air was 14 dm^3^/min. Standard solutions (Mg^2+^, Zn^2+^–respectively 100 and 5.0 mg/L) were prepared from 1000 mg/L standard solution (Merck, Bilerica, MA, USA).

#### 2.3.3. Organic Acid and Carbohydrate Profile Analysis

Specific enzymatic kits (Megazyme, Wicklow, Ireland) were used to determine the concentrations of L-lactic acid (kit K-LATE 06/18), acetic acid (kit K-ACET 04/18), L -malic acid (kit K-LMALQR 03/18), succinic acid (kit K-SUCC 06/18) and carbohydrates such as D-glucose, D-fructose, maltose and sucrose (K-FRGLQR 02/17, K-MASUG 11/16) according to the manufacturer’s instructions. LA content was determined daily during lactic fermentation and all acids were measured at the end of the process. The carbohydrate concentration was measured before (in the wort) and after fermentation (in beer). The concentrations of D-glucose and D-fructose were also measured after LA fermentation.

#### 2.3.4. Analysis of Aroma Compounds by Solid-Phase Microextraction–Gas Chromatography/Mass Spectrometry

A 1 mL aliquot of saturated saline with an internal standard solution (5 mg/L 4-methyl-2-pentanol and 0.05 mg/L ethyl nonanoate, Sigma-Aldrich, St. Louis, MO, USA) and a 1 mL sample of beer were added into a 10 mL vial. The SPME device (Supelco Inc., Bellefonte, PA, USA) containing PDMS (100 μm) fibres was first conditioned by placing it in the GC injector port at 250 °C for 1 h. For sampling, the fibre was inserted into the headspace under stirring (300 rpm) for 30 min at 40 °C. Subsequently, the SPME device was introduced into the injector port of an Agilent Technologies 7890B chromatograph system equipped with a LECO Pegasus high-throughput time-of-flight mass spectrometry and kept in the inlet for 3 min. The SPME process was automated using a Gerstel MultiPurpose Sampler (MPS).

The tested components were separated using a Restek™ Rxi-1ms Capillary Column (Crossbond 100% dimethyl polysiloxane, 30 m × 0.53 mm × 0.5 μm). The column was heated using the following temperature program: 40 °C increasing to 230 °C, over 3 min at a rate of 8 °C/min, then held at a constant temperature of 230 °C for 9 min. Carrier gas: helium at 1.0 mL/min constant flow. Electron ionisation mass spectrometry energy: 70 eV; ion source temperature and connection parts: 250 °C. Analyte transfer was performed in splitless mode; the mass spectrometry detector was set to scan mode from m/z = 40 to m/z = 400.

Compounds were identified using mass spectral libraries and linear retention indices, calculated from a series of n-alkanes from C6 to C30. The qualitative and quantitative identification of the aroma compounds (Ethyl Acetate; 1-Propanol, 2-methyl-; 1-Butanol, 3-methyl-; 1-Butanol, 2-methyl-; Butanoic acid, ethyl ester; 2,3-Butanediol; 1-Hexanol; 1-Butanol, 3-methyl-, acetate; 2-Furanmethanol, Butyrolactone; Isomaltol; Hexanoic acid; Hexanoic acid, ethyl ester; 2-Ethyl-1-hexanol; Furaneol; 1-Octanol; Phenylethyl Alcohol; Octanoic acid; α-Terpineol; Octanoic acid, ethyl ester; Benzothiazole; Octanoic acid, 2-methyl-, ethyl ester; Acetic acid, 2-phenylethyl ester; Geraniol; 1-Decanol, γ-Nonalactone; n-Decanoic acid; 1-Undecanol; β-Damascenone; Decanoic acid, ethyl ester; Dodecanoic acid; Dodecanoic acid, ethyl ester; Benzophenone, Benzyl benzoate; Tetradecanoic acid, ethyl ester; Hexadecanoic acid, ethyl ester; Sigma-Aldrich, St. Louis, MO, USA) was based on the comparison of retention times and peak areas from the sample and standard chromatograms. Other detected components were determined semi-quantitatively by comparing the relative peak area of each identified compound, according to the National Institute of Standards and Technology Database (http://webbook.nist.gov/chemistry/), to that of the internal standard.

### 2.4. Statistical Analysis

Values are given as mean ± standard deviation. Data were analysed using a two-way analysis of variance (ANOVA) to test the significance level of each factor’s influence. The differences in each parameter were analysed separately using Duncan’s Multiple Range Test (DMRT) (Statistica v. 10, StatSoft Inc., Krakow, Poland). For both ANOVA and DMRT, values < 0.05 were considered statistically significant (*p* < 0.05).

## 3. Results and Discussions

### 3.1. Organic Acid Production and pH

The LA concentration and pH are the main characteristics used to classify a sour beer. According to the literature, sour beers should contain LA in the range of 3–6 g/L and have a pH of 3.3–3.9 [1,2,20] but some sour beers containing lower levels of LA (2.1–3.4 g/L) have also been reported [21].

**Figure 1:**
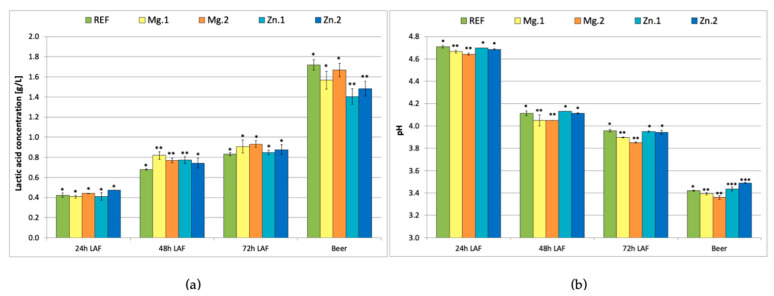
L-Lactic acid concentration (**a**) and pH (**b**) of fermenting wort measured every 24 h during lactic acid fermentation (LAF) and in the beer; REF: reference sample (without supplementation); Mg.1, Mg.2: wort supplemented with magnesium at two different levels; Zn.1, Zn.2: wort supplemented with zinc at two different levels. Bars show the average of three replicates ± standard deviation. *, ** and *** indicate homogeneous groups (*p* < 0.05) within the samples analysed at a certain timepoint.

Figure 1 shows the LA concentration and pH respectively, measured every 24 h during the LA fermentation and in the produced beer. The LA content increased during LA fermentation to reach the maximum level of 0.82–0.90 g/L in all samples (Figure 1a, 72 h LAF). After 24 h, there were no significant differences between the reference and the supplemented samples (the lactic acid content was ca. 0.4 g/L). After 48 h, the LA content had increased to 0.68 g/L in the reference sample and 0.75–0.82 g/L in the supplemented samples. Thus, the total amount of lactic acid produced by the bacteria was the same in all trials, but supplementation with either magnesium or zinc ions shortened the time required (48 h vs. 72 h).

A further increase in the lactic acid content was observed after ethanol fermentation. The highest levels were obtained in the reference sample and the samples supplemented with magnesium ions (1.55–1.65 g/L), while the lowest levels were observed in the samples supplemented with zinc ions (1.40–1.50 g/L). As expected, zinc ions enhanced the fermentation performed by yeast but did not improve lactic acid production. McLeod and Snell [10] showed that zinc can be toxic to some LAB if the growth media does not contain sufficient manganese. The requirement for zinc is around 10 times lower in the case of LAB as compared to yeast [11]. Therefore, the addition of zinc did not enhance LAB fermentation but improved ethanol production by yeast. According to the literature, the L-LA content in green beer reaches ca. 1 g/L when *L. brevis* is used [1,5,22], which was concordant with our results.

Considering that the rate of LA production was enhanced in the wort samples supplemented with either magnesium or zinc ions, but zinc supplementation inhibited the production of LA during the mixed fermentation performed by bacteria and yeast, it is concluded that zinc supplementation is not recommended in sour beer production.

The addition of magnesium ions to the wort had a positive influence in the performance of the LAB as shown by the rate of pH decrease of the wort (Figure 1b). Supplementation with Mg^2+^ (at both 60 and 120 mg/L) resulted in a lower wort pH measured 72 h after addition of LAB (pH 3.90 and 3.85, respectively) as compared to the reference wort (pH 3.95). Consequently, the beers produced with Mg^2+^ supplementation (after alcoholic fermentation) had lower pH (<3.4) than the reference beer (>3.4). On the contrary, the beers produced from the wort supplemented with zinc ions had slightly higher pH (3.45 and 3.5) than the reference beer.

**Figure 2:**
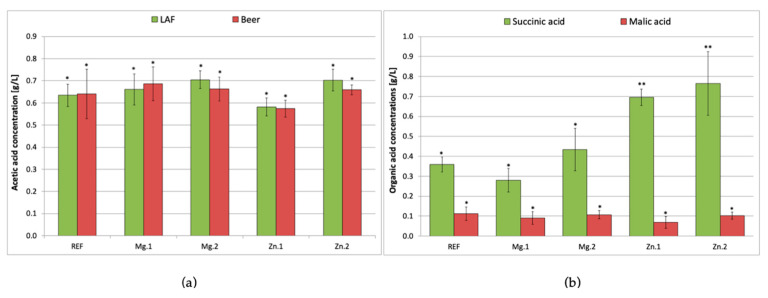
Acetic acid concentration (**a**) after 72 h of lactic acid (LAF) and ethanol fermentation (beer), and succinic and malic acid (**b**) in beer; REF: reference sample (without supplementation); Mg.1, Mg.2: wort supplemented with magnesium at two different levels; Zn.1, Zn.2: wort supplemented with zinc at two different levels. Bars show the average of three replicates ± standard deviation. * and ** indicate homogeneous groups within the same compound (*p* < 0.05).

In addition to LA (the main metabolite), *L. brevis* also produced other organic acids. Their concentrations are shown in Figure 2.

According to the literature, *L. brevis* is able to produce 0.2–0.4 g/L of acetic acid [1,22,23,24], while the detectability threshold in beer is 0.2 g/L [25]. In the current work significantly higher acetic acid concentrations 0.6–0.7 g/L were obtained (Figure 2a). *L. brevis* produces CO_2_ and ethanol or acetic acid in addition to LA. The proportions of these metabolites depend on the composition of the medium: if it is rich in glucose, LA is the main metabolite, whereas if the medium is low in glucose or if maltose/galactose dominate, the amounts of acetic acid/ethanol and CO_2_ produced increase, while LA biosynthesis decreases [23]. This phenomenon was confirmed in our research. The main carbohydrate in malt wort is maltose (approximately 50 g/L), while glucose accounts for only 12% of the carbohydrates (7–8 g/L), which is why higher concentrations of acetic acid were obtained than the values reported in the literature. After alcoholic fermentation, the amount of acetic acid did not change significantly in any of the samples. On the contrary, the l-LA content more than doubled after alcoholic fermentation (Figure 1a).

Succinic and malic acids are also produced in the course of fermentation. According to the literature, LAB can produce 0.2–0.8 g/L of succinic acid but this is very strain-dependent [5,22]. Figure 2b shows the succinic and malic acid contents in the beer. In the samples supplemented with zinc ions (Zn.1 and Zn.2), the succinic acid content was almost double that in the reference (0.36 and 0.77 g/L, respectively). Tyrell [26] studied various parameters affecting the formation of succinic acid in beer and concluded that aeration, extract content in the wort, pH and fermentation temperature are the major ones. Our research proves that zinc ions can be also taken into account as a factor contributing to the succinic acid concentration in beer. Nevertheless, a high concentration of succinic acid in beer is not desirable because it stimulates the secretion of stomach acids, causing indigestion and lowering the drinkability of the beer [27]. Supplementation of the wort with magnesium ions did not lead to a significant difference from the reference values (0.28–0.43 g/L).

Malic acid is produced during the malting process by endogenous microorganisms [28]. In malt wort, malic acid is present at low levels of around 0.4 g/L, decreasing to around 0.1 g/L after lactic fermentation [5]. Bacteria transform malic acid as a result of malolactic fermentation, and the intensity of this process depends on the bacterial strain [6]. In the present study, there were no significant differences in malic acid content across all samples (0.07–0.1 g/L).

### 3.2. Carbohydrate Use

The four fermentable carbohydrates and their initial concentrations in wort were: maltose (49.5 ± 1.3 g/L), glucose (7.7 ± 1.6 g/L) fructose (3.0 ± 0.6 g/L) and saccharose (1.8 ± 0.3 g/L). Maltose is the most abundant carbon source in malt wort, and it is fully used by yeast during fermentation [29]. However, this is not the case for *Lactobacillus*-according to some studies, only about 4% of maltose contained in the wort is used by *Lactobacillus* [1,5]. The crucial carbohydrates for LAB are glucose and fructose, with their order of uptake depending mainly on the strain e.g., *L. amylolyticus* and *L. plantarum* show a preference towards glucose, while *L. brevis* mainly ferments fructose [5,24]. Therefore, we focused on the use of those carbohydrates (Table 2).

After 72 h of lactic fermentation, fructose was fully used, regardless of the supplementation variant. There have been no reports to date proving that fructose is an essential carbohydrate for LAB growth, but it can be supposed that its higher concentration in the medium could contribute to an increase in the bacteria’s metabolic activity. The use of fructose as an electron acceptor gives the LAB an additional ATP gain without ATP expenditure when converting acetyl-P to acetate [30]. Therefore, it is suggested that the production of fructose-rich wort (e.g., with the use of unmalted raw materials) may be a good solution for the production of sour beers, especially when *Lactobacillus brevis* is used.

Glucose is the main carbohydrate fermented by LAB according to many studies [1,23,24]. In work presented by Nsogning et al. [5], LAB used 20–50% of the glucose present in the wort. The strain *L. amylolyticus* La.TL3 has the highest preference for glucose (using 6 g/L), while the lowest preference is expressed by *L. brevis Lb.986* (2 g/L). In the current work, glucose was only slightly processed at about 1 g/L. Interestingly, an increase in glucose content was noted in samples supplemented with zinc after lactic fermentation (8.06 g/L in Z.2 vs. 6.68 g/L in REF), which could be associated with the action of enzymes released from the cytoplasm after cell death that may have broken down sucrose into glucose and fructose. A similar relationship was observed in research by Peyer [1], where he noted an increase in glucose content in the medium during *L. brevis* R2Δ fermentation while hydrolysing sucrose. Nsogning et al. [5] noted an increase in fructose content during LAB fermentation, with a high percentage of dead cells (60–80%).

Sucrose is not considered as an essential carbohydrate for LAB fermentation, especially for heterofermentative strains [5], so we did not monitor its concentration beyond the 72 h of lactic fermentation. However, after alcoholic fermentation, all the monosaccharides present in the wort had been completely consumed (Table 2), while around 93% of the maltose was used, regardless of the experiment variant (*p* = 0.9). Assume that, supplementation with Mg^2+^ and Zn^2+^ had no effect on carbohydrate use by bacteria and yeast, but slightly affected the ethanol content and the real extract in beer (Table 3). The addition of zinc positively influenced alcoholic fermentation, resulting in a slightly higher ethanol concentration compared to the reference.

### 3.3. Aroma Compounds

According to the literature, the main volatile compounds produced by LAB in malt-based fermented beverages are principally associated with carbohydrate metabolism (esters, aldehydes and ketones) and amino acid metabolism (higher alcohols and aldehydes) [31,32]. They may arise as a result of malt wort fermentation or the conversion of precursors derived from malt wort by LAB enzymes [32]. Both LAB and yeast produce many aroma compounds during the fermentation of malt wort. Yeast produces mainly higher alcohols, esters, aldehydes and fatty acids, while LAB produce organic acids and aldehydes [1,33].

It was proved that the initial concentration of metal ions in wort influences the concentration of some aroma compounds in beer. Zinc ions have a significant impact on the biosynthesis of higher alcohols, carboxylic acids and terpenes; their content was higher by more than double in samples supplemented with zinc ions, as compared to the reference and to the magnesium supplemented samples (Table 4).

Fatty acids have a beneficial effect on yeast growth during fermentation as well as a negative effect on the foam and sensory stability of beer [34]. In general, long-chain fatty acids originate from raw materials, whereas short-chain acids are produced by yeast during fermentation [35]. The high levels of fatty acids seen in the samples supplemented with zinc ions (Table 4) may be caused by increased yeast metabolism (as in the case of higher alcohols) or a LAB response to stress factors (oxidative, osmotic, acid or heat) during fermentation [36]. Usually, high levels of hexanoic, octanoic and n-decanoic acid in beer are not desirable because they cause an unpleasant, rancid smell and taste [34].

Geraniol, α-terpineol and β-damascenone (Table 4) are always associated with hop oils but they have also been reported to be present in un-hopped wort [37]. They may be formed during malolactic fermentation by glycosidase and arabinosidase from precursors, or thermal cleavage by heat treatment under acidic conditions [32]. In the trials supplemented with zinc ions, we observed 7–12 times higher concentrations of geraniol and α-terpineol compared to the reference. It is not entirely clear why the increased zinc supplementation resulted in higher amounts of these two terpenes. Metal ions, such as manganese, have been reported to increase the beta-glucosidase activity by as much as twofold [38]; perhaps Zn^2+^ also plays an important role in the activity of glucosidase, which is responsible for releasing terpenoids from the bound form [24]. Nevertheless, the values are below the flavour threshold of 1 mg/L [39].

Furthermore, the contents of higher alcohols, such as 1-Propanol, 2-methyl-; 1-Butanol, 3-methyl-; 1-Butanol, 2-methyl- and Phenylethyl Alcohol were significantly higher in the trials with Zn^2+^ supplementation as compared to the reference sample (Table 4). Similar results were obtained by Gutierrez [40], who revealed lower production of 1-Propanol, 2-methyl and 1-Butanol, 3-methyl- with zinc deficiency in the fermentation medium. This can be explained by the essential role played by zinc ions in the operation of several enzymes, such as alcohol dehydrogenase, aldolase and alkaline phosphatase [11]. Higher alcohols originate from the metabolism of amino acids. In yeast, the synthesis of branched-chain amino acids is regulated by the expression of the genes *ILV2*, *ILV3* and *BAT2*. These genes were found to be downregulated in zinc-limited yeast growth in the presence or absence of oxygen [41].

In work, the ester concentration was not influenced by the initial zinc and magnesium ion content. This may be because ionic supplementation of wort does not affect the production of amino acids or fatty acids, which are precursors of esters, as the ions are not cofactors for enzymes involved in the production of esters (i.e., esterase, alcohol acetyltransferase) [42]. Biosynthesis of esters by LAB is based on the transfer of a fatty acyl group from acylglycerol and acyl-CoA to an alcohol (Scheme 1). However, as a result of the low ethanol production by LAB (<0.5% *v*/*v*) [2], lactic fermentation does not favourably affect esterification, which is generally desirable because esters give a sweet, fruity taste. LA and acetic acid (the main organic acids produced during lactic acid fermentation) may be involved in the formation of the esters ethyl lactate and ethyl acetate, respectively [32].

### 3.4. Metal Ion Release by LAB and Yeast

**Figure 3:**
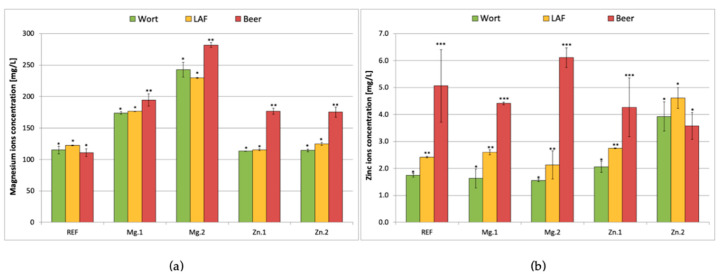
Magnesium (**a**) and zinc (**b**) concentration in the wort before fermentation, after lactic acid fermentation and in the produced beer (after lactic acid and alcoholic fermentation) depending on the initial concentrations of magnesium and zinc ions: REF: reference sample (without supplementation); Mg.1, Mg.2: supplemented with magnesium at two different levels; Zn.1, Zn.2: supplemented with zinc at two different levels. Results are the means of three replicates and the error bars are ± SD, *, ** and *** indicate homogeneous groups within the same trial (*p* < 0.05).

Figure 3 shows the concentration of magnesium (a) and zinc (b) ions in the wort during the mixed fermentation depending on the initial content of this ions in the wort. The lactic acid fermentation did not cause any changes in the magnesium content in the wort, which indicates that the LAB did not take up or release magnesium during the 72 h of the process. On the contrary, yeast released magnesium, which resulted in a slight increase in the magnesium content.

In all of the trials supplemented with metal ions (either magnesium or zinc), the yeast released magnesium into the medium at the end of the process. In the samples with magnesium supplementation, the release was less pronounced than in the case of the zinc supplementation. In the former one (trial Mg.1, 60 mg/L supplementation) the increase in the Mg^2+^ content was ca. 10% (from 165 to 190 mg/L), while in the case of trial Mg.2 (120 mg/L supplementation) it was ca. 20% (from 225 to 280 mg/L). In the latter case (zinc supplemented samples), the increase in the magnesium ion in the beer after fermentation was much higher, reaching 50% (from 120 to 175 mg/L in both the 0.4 and 2 mg/L Zn^2+^ supplementation trials). Similar trends were observed in study Poreda et al. [43], which proved that increased content of zinc ions in wort can block the uptake of magnesium ions by the yeast. The current results show that in the case of *L. brevis* strain the uptake and release of magnesium ions is not affected by the zinc ion content.

The pattern of release of zinc ions during subsequent LA and alcoholic fermentation was different from that observed for magnesium (Figure 3b). First of all, there was already an increase in the zinc ion concentration in the wort after performing the lactic acid fermentation (in all treatments except for the one with the highest level of Zn^2+^ supplementation). It is worth noting that the highest release of zinc ions (Zn^2+^ increased from 2 to 6 mg/L in the wort) was observed in the trial with the highest supplementation of magnesium ions (120 mg/L). In the reference sample and the sample with 60 mg/L magnesium supplementation, the zinc ion concentration in the wort increased from around 2.5 mg/L up to ca. 4.5–5 mg/L. In the trial with Zn^2+^ supplementation at 2 mg/L, neither the LAB nor the yeast cells released zinc ions into the wort. It seems that the concentration in the wort was high enough to block any leakage of Zn^2+^ ions out of the cells. Based on the results two conclusions can be drawn. First of all, the release of zinc ions from both LAB and yeast cells proceeds via passive transport dependent on the Zn^2+^ concentration gradient. The higher the content of zinc ions in the wort, the fewer ions were released by the microbes. Secondly, the rate of zinc ion release from the yeast cells depends not only on the zinc ion content in wort but also on the magnesium ion content. The more magnesium that was present in the wort (Mg.2), the more zinc ions were released out of the cell. A similar trend was observed in earlier studies, which proved that at levels above 150 mg/L, magnesium ions effectively limit zinc bioaccumulation in yeast biomass [43].

## 4. Conclusions

The objective of this study was to determine how metal ion (magnesium and zinc) supplementation affects the metabolism of *Lactobacillus brevis WLP672* in brewers’ wort, the production of organic acids and volatile compounds, and the rate of sour beer fermentation.

Supplementation of wort with magnesium resulted in a slightly faster decrease in the pH during the fermentation and an overall the lowest pH of the sour beer. The differences, though statistically significant, may be negligible at the industrial production, so more work is necessary in this field to further increase the rate of pH decrease during LAB fermentation. Additionally, the concentration of lactic acid after 48 h of LAB fermentation was higher in the samples with Mg^2+^ supplementation, compared to the reference sample. On the other hand, zinc ions supplementation resulted in a higher pH and lower content of lactic acid in the sour beer. It is an important finding, as zinc supplementation is a standard procedure in many breweries, and it is worth considering reducing or abandoning Zn^2+^ supplementation when producing wort for sour beer. We also found almost twofold higher concentrations of succinic acid in the Zn^2+^ supplemented samples than in the reference sample or the Mg^2+^ supplemented samples. High concentration of succinic acid in beer is not desirable, as it results in beer with lower drinkability. Regarding aroma compounds, supplemental magnesium increased the concentrations of 1-Propanol, 2-methyl-; 1-Butanol, 3-methyl-; Hexanoic acid, Octanoic acid, ethyl ester; Benzothiazole and Benzyl Benzoate, while zinc most of all enhanced the synthesis of carboxylic acids and terpenes. Supplementation with magnesium and zinc had no effect on carbohydrate use but slightly affected the ethanol content and the real extract in beer. The addition of zinc positively influenced alcoholic fermentation, resulting in a slightly higher alcohol concentration compared to the reference.

This work will contribute to the brewing industry by providing new knowledge about the impact of magnesium or zinc supplementation on the lactic acid and alcoholic fermentation. It will also help to understand the relation between ionic content of wort and the aroma profile of beer, which can lead to improvement of sour beer quality. Further work is needed to evaluate how changes in other wort characteristics (carbohydrates profile, free amino nitrogen content) influence the lactic acid production and sensory quality of sour beers.

## Figures and Tables

**Table 1:** Initial magnesium and zinc ion contents [mg/L] in the different wort samples before fermenting.

	REF	Mg.1	Mg.2	Zn.1	Zn.2
Mg^2+^	115.0 ± 6.0	173.7 ± 2.0	242.7 ± 11.6	113.4 ± 0.2	114.4 ± 2.2
Zn^2+^	1.7 ± 0.1	1.6 ± 0.1	1.6 ± 0.0	2.1 ± 0.2	3.9 ± 0.5

**Table 2:** Concentration of carbohydrates [g/L] in malt wort before fermentation, after 72 h of lactic acid fermentation and after alcoholic fermentation depending on the initial concentration of magnesium and zinc ions: REF: reference sample (without supplementation); Mg.1, Mg.2: wort supplemented with magnesium at two different levels; Zn.1, Zn.2: wort supplemented with zinc at two different levels.

Wort						After Lactic Acid Fermentation					After Ethanol Fermentation				
Carbohydrate [g/L]	REF	Mg.1	Mg.2	Zn.1	Zn.2	REF	Mg.1	Mg.2	Zn.1	Zn.2	REF	Mg.1	Mg.2	Zn.1	Zn.2
Glucose	7.7 ± 1.6					6.7 ± 0.5 *	7.1 ± 0.8 *	6.7 ± 0.2 *	7.1 ± 0.1 *	8.1 ± 0.3 **	0.1 ± 0.0 *	0.1 ± 0.0 *	0.1 ± 0.0 *	0.1 ± 0.0 *	0.1 ± 0.0 *
Fructose	3.0 ± 0.6					0.0 ± 0.0					0.0 ± 0.0				
Sucrose	1.8 ± 0.3										0.0 ± 0.0				
Maltose	49.5 ± 1.3										3.3 ± 0.5 *	3.6 ± 0.4 *	3.4 ± 0.4 *	3.6 ± 0.4 *	3.6 ± 0.6 *

Results are presented as the mean of three replicates ± standard deviation, * indicates that the sample is not significantly different from the reference and ** indicates that the sample is significantly different from the reference sample (*p* < 0.05).

**Table 3:** Ethanol and real extract content in beer depending on the initial concentrations of magnesium (Mg.1 Mg.2) and zinc (Zn.1 and Zn.2) ions in wort. REF-beer obtained from wort without supplementation.

	REF	Mg.1	Mg.2	Zn.1	Zn.1
Ethanol [% *v*/*v*]	3.9 ± 0.0 *	4.0 ± 0.0 **	3.9 ± 0.0 *	4.0 ± 0.0 **	4.2 ± 0.0 **
Real extract [% *w*/*w*]	6.2 ± 0.0 *	6.5 ± 0.1 **	6.4 ± 0.0 **	6.3 ± 0.0 *	6.6 ± 0.0 **

Results are presented as the mean of three replicates ± standard deviation, * indicates that the sample is not significantly different from the reference and ** indicates that the sample is significantly different from the reference sample (*p* < 0.05).

**Table 4:** The amounts of aroma compounds (higher alcohols, esters, fatty acids and terpenes) in sour beer produced during subsequent lactic acid and alcoholic fermentation of brewers’ wort depending on the initial concentrations of magnesium (Mg.1 Mg.2) and zinc (Zn.1 and Zn.2) ions. REF-beer obtained from wort without supplementation.

		Sample [µg/L]					
	LRI ^2^	REF	Mg.1	Mg.2	Zn.1	Zn.2	Sig. ^1^
**Esters**							
Ethyl acetate	614	2716 ^a^	3224 ^a^	2740 ^a^	4099 ^b^	2733 ^a^	*
Butanoic acid, ethyl ester ^3^	789	0.3 ^b^	0.4 ^b^	0.5 ^c^	0.0 ^a^	0.0 ^a^	***
1-Butanol, 3-methyl-, acetate	872	16.7 ^d^	9.5 ^c^	6.6 ^b^	3.7 ^a^	2.7 ^a^	***
Hexanoic acid, ethyl ester	986	2.8 ^b^	2.5 ^b^	2.1 ^ab^	1.6 ^a^	1.9 ^ab^	*
Octanoic acid, ethyl ester	1180	34.6 ^a^	53.6 ^b^	61.9 ^b^	54.6 ^b^	57.9 ^b^	*
Octanoic acid, 2-methyl-, ethyl ester ^3^	1209	69.3 ^b^	33.4 ^a^	26.3 ^a^	30.6 ^a^	30.1 ^a^	***
Acetic acid, 2-phenylethyl ester	1228	64.2 ^c^	63.2 ^c^	46.8 ^b^	37.4 ^ab^	30.5 ^a^	***
Decanoic acid, ethyl ester	1397	11.8 ^b^	11.4 ^b^	11.7 ^b^	7.9 ^ab^	6.9 ^a^	*
Dodecanoic acid, ethyl ester	1581	6.3 ^a^	8.1 ^b^	6.2 ^a^	6.0 ^a^	4.7 ^a^	*
Benzyl Benzoate	1750	1.3 ^a^	3.8 ^b^	3.8 ^b^	10.7 ^c^	13.2 ^d^	***
Tetradecanoic acid, ethyl ester ^3^	1790	0.8 ^a^	2.6 ^ab^	3.5 ^b^	12.6 ^c^	14.7 ^d^	***
Hexadecanoic acid, ethyl ester ^3^	1990	0.6 ^a^	2.0 ^a^	2.6 ^a^	12.2 ^b^	14.7 ^c^	***
**Higher alcohols**							
1-Propanol, 2-methyl-	617	433.6 ^a^	699.2 ^b^	725.5 ^b^	977.8 ^c^	1046.8 ^c^	***
1-Butanol, 3-methyl-	723	13,787 ^a^	21,007 ^bc^	19,298 ^b^	24,456 ^c^	29,083 ^d^	***
1-Butanol, 2-methyl-	740	4984 ^a^	6818 ^b^	5173 ^a^	6093 ^ab^	7235 ^b^	***
2,3-Butanediol	768	1663 ^bc^	1861 ^c^	1322 ^b^	838 ^a^	553 ^a^	***
1-Hexanol	865	23.7 ^a^	36.0 ^b^	25.8 ^a^	24.9 ^a^	23.8 ^a^	*
2-Furanmethanol ^3^	880	48.8 ^a^	130.1 ^c^	73.1 ^ab^	75.4 ^b^	73.3 ^a b^	***
Isomaltol ^3^	975	48.5 ^a^	104.4 ^c^	72.1 ^ab^	77.3 ^b^	146.0 ^d^	***
2-Ethyl-1-hexanol ^3^	1020	99.9 ^b^	97.3 ^b^	78.1 ^a^	73.2 ^a^	64.5 ^a^	***
Furaneol ^3^	1057	35.8	43.3	41.2	40.1	42.3	ns
1-Octanol ^3^	1070	19.9	21.5	16.9	14.7	13.6	ns
Phenylethyl Alcohol	1084	3320.3 ^a^	4966.0 ^b^	3233.5 ^a^	3193.6 ^a^	5582.0 ^b^	***
1-Decanol ^3^	1272	192.3 ^a^	179.6 ^a^	312.3 ^c^	244.2 ^b^	159.3 ^a^	***
1-Undecanol ^3^	1374	44.2	59.0	56.3	57.5	73.3	ns
**Fatty acids**							
Hexanoic acid	982	2.7 ^a^	7.2 ^b^	6.9 ^b^	19.7 ^c^	27.6 ^d^	***
Octanoic acid	1160	62.2 ^a^	173.5 ^a^	184.2 ^a^	584.3 ^b^	1102.2 ^c^	***
n-Decanoic acid	1368	3.9 ^a^	9.5 ^a^	11.2 ^a^	30.7 ^b^	36.8 ^b^	***
Dodecanoic acid	1568	0.6 ^a^	2.0 ^b^	1.4 ^ab^	4.3 ^c^	6.8 ^d^	***
**Terpenes**							
α-Terpineol	1171	13.0 ^a^	28.6 ^ab^	50.3 ^b^	96.8 ^c^	159.1 ^d^	***
Geraniol	1257	27.6 ^a^	59.2 ^ab^	91.5 ^b^	201.1 ^c^	248.0 ^d^	***
β-Damascenone ^3^	1386	29.2	30.6	32.6	31.6	30.5	ns
**Lactones**							
Butyrolactone ^3^	916	79.7	72.0	80.9	73.3	42.9	ns
γ-Nonalactone ^3^	1360	64.4	75.2	62.2	54.2	57.4	ns
**Other compounds**							
Benzothiazole ^3^	1186	247.8 ^a^	437.2 ^b^	592.8 ^c^	586.1 ^c^	772.4 ^d^	***
Benzophenone ^3^	1610	5.2	6.5	4.9	4.8	5.0	ns

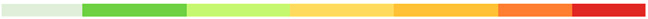
The colours (from light to dark) indicate increasing values within the same volatile compound. ^1^ Sig.: significance; * and *** indicate significance at the 5, 1 and 0.5% levels by least significant difference; ns: not significant. Values with different superscript roman letters (^a–d^) in the same row are significantly different according to the Duncan test (*p* < 0.05) ^2^ LRI: Linear retention index ^3^ Determined semi-quantitatively by measuring the relative peak area of each identified compound, according to the NIST database, and comparing it to that of the internal standard.
